# School-based intervention to improve the mental health of low-income, secondary school students in Santiago, Chile (YPSA): study protocol for a randomized controlled trial

**DOI:** 10.1186/1745-6215-12-49

**Published:** 2011-02-19

**Authors:** Ricardo Araya, Alan A Montgomery, Rosemarie Fritsch, David Gunnell, Paul Stallard, Sian Noble, Vania Martinez, Sergio Barroilhet, Paul Vohringer, Viviana Guajardo, Felix Cova, Jorge Gaete, Alejandro Gomez, Graciela Rojas

**Affiliations:** 1 University of Bristol, School of Social and Community Medicine, Oakfield Grove, Bristol BS8 2BN, UK; 2 University of Bristol, School of Social and Community Medicine, Canynge Hall, 39 Whatley Road, Bristol BS8 2PS, UK; 3 Universidad de Chile, Departamento de Psiquiatría, Avda. La Paz 1003, Santiago, Chile; 4 University of Bath, Department for Health, Bath BA2 7AY, UK; 5 Universidad de Concepción, Facultad de Ciencias Sociales, Dpto. Psicologia, Barrio Universitario s/n, Concepcion, Chile; 6 Universidad de Los Andes, Escuela de Piscología, San Carlos de Apoquindo 2200, Santiago, Chile

## Abstract

**Background:**

Depression is common and can have devastating effects on the life of adolescents. Psychological interventions are the first-line for treating or preventing depression among adolescents. This proposal aims to evaluate a school-based, universal psychological intervention to reduce depressive symptoms among student's aged 13-14 attending municipal state secondary schools in Santiago, Chile.

**Study design:**

This is a cluster randomised controlled trial with schools as the main clusters. We compared this intervention with a control group in a study involving 22 schools, 66 classes and approximately 2,600 students. Students in the active schools attended 11 weekly and 3 booster sessions of an intervention based on cognitive-behavioural models. The control schools received their usual but enhanced counselling sessions currently included in their curriculum. Mean depression scores and indicators of levels of functioning were assessed at 3 and 12 months after the completion of the intervention in order to assess the effectiveness of the intervention. Direct and indirect costs were measured in both groups to assess the cost-effectiveness of this intervention.

**Discussion:**

As far as we are aware this is the first cluster randomised controlled trial of a school intervention for depression among adolescents outside the Western world.

**Trial Registration:**

ISRCTN19466209

## Background

Depression is a common and disabling condition affecting people of all ages and races[1]. The prevalence of depression is high among adults[2-5] and children[6,7] in Latin America. Depression can have devastating effects on the life of adolescents affecting school performance, increasing antisocial behaviour, self-harm and suicide, and impairing social interactions[8-13].

Psychological interventions are the first-line treatment for most adolescents with depressive disorders[14] and several systematic reviews have shown their efficacy[15,16]. Two main psychotherapeutic modalities have been used: Cognitive-Behavioural Therapy (CBT) and Interpersonal Psychotherapy (IPT) with both showing promising results[15-18]. The evidence to show the efficacy of psychological interventions to prevent adolescent depression is less consistent but still encouraging.

Depressive symptoms are strong predictors of the onset of depressive episodes [12,19], symptoms are strongly associated with impairment[11], and depression can be conceived as a dimensional rather than a categorical construct. Thus, excluding adolescents with depressive symptoms of moderate or lesser severity from preventive interventions seems unwarranted. The more traditional and narrow definition of preventive interventions, as those preventing new cases, has been replaced by a more pragmatic one that includes interventions to reduce existing symptoms among adolescents with sub-threshold clinical conditions. Preventative interventions are classified as 'universal' or 'targeted'[20]. Whilst 'universal' interventions cover the entire population at risk, 'targeted' interventions are given to normal adolescents with a known risk factor (selective) or to teens with sub-threshold depressive symptoms (indicated).

Most studies of interventions to prevent depression have taken place in schools, which seems an excellent milieu to introduce universal, large-scale interventions. A systematic review of preventative interventions for depression in children and adolescents[21] found 10 'universal' studies. The majority of these studies were small and methodologically poor, involved highly selected samples of variable ages, and tested very different interventions. A valid synthesis of these findings is therefore challenging. This review also excluded studies of individuals meeting depressive disorder criteria; effectively removing most studies of youngsters with mild and/or moderate depression. Notwithstanding these limitations two of the largest studies included in this review[22,23] and three recent universal school-based studies have shown more favourable results. In Germany, students receiving a 10-session course based on self-efficacy and CBT models presented a symptom reduction 0.5 standard deviations (SD) lower than those in a control group[24]. In New Zealand, students receiving 11 CBT sessions fared better than a placebo-group immediately after the intervention but there were no differences at 18 months[25]. Finally an Australian study compared a CBT intervention delivered to: a) all students, b) only those with high symptom scores, or c) a control group. All groups improved similarly with a reduction in symptom scores between 0.5-1 SD at 12 months[26]. Most studies show short-term effects that tend to fade away with time, interventions are usually teacher-led, rarely include booster sessions, and there are often significant losses over follow-up[23,27]. It is also worth noting that most universal, school-based interventions have been based on CBT models. There are no published universal studies using IPT in schools. The only small study comparing the efficacy of individual IPT and CBT for depressed adolescents found equally good post-treatment results for both but only CBT related improvements were maintained at 3 months[18].

Targeted ("indicated") interventions have shown larger effect sizes than universal interventions[21]. This is probably related to the increased severity of symptoms of participants in targeted interventions and a possible 'floor-effect' (unchanged 'normals') affecting universal interventions more markedly. Targeted interventions are also more intensive and delivered to smaller groups. However such interventions present problems with recruitment and retention[28-30] and often exclude students who would benefit. Merry et al concluded that 'given the practical difficulties inherent in implementing a targeted programme, pursuing the implementation of universal depression programmes is warranted. In prevention programmes, the asymptomatic group often yields more cases than the sub-syndromal group because it is a larger group'[21].

Several issues remain a challenge in depression preventative research such as designing better interventions, following participants for long enough and most importantly replicating studies in non-western populations. Almost all the studies reviewed were carried out in the Western world with relatively affluent populations and thus this evidence cannot be easily generalised to the developing world.

There is an urgent need to develop effective and affordable programmes to help young adolescents cope with emotional problems in resource-poor settings. This must become a priority especially when it is known that preventing, delaying or early treatment of depression can have profound implications on the consequences of this disabling disorder across the life course[31,32].

Chile is a middle-income country ranked amongst the most unequal countries with respect to income[33]. Depression is common among children and adolescents in Chile[34] with a point-prevalence around 10% in the general population and higher levels among low-income children[3]. We found that the prevalence of common mental disorders at age 17 almost doubled that at age 15 (12% vs 27%) in a large household survey(2). Low income people are significantly less likely to receive any help for their emotional problems[35]. Almost all (98%) secondary students from low income families attend state funded secondary schools [36]. Schools offer a great opportunity to access poor families and to help youngsters in need before their emotional problems become entrenched and the consequences fully manifested.

This proposal is a continuation of our previous successful randomised controlled trials in Chile[37-39]. The intervention we tested in our first trial for the treatment of depressed low-income women in primary care led to the introduction of a national programme[40]. The current proposal builds on the knowledge and experience acquired in these previous trials and extends our approaches beyond the health sector, with the aim of developing links with the educational sector. In recognition of the benefits of prevention and the potentially lifelong benefits on emotional health or skills learnt in childhood, our focus is on adolescents aged 13-15.

## Methods and design

### Aims and hypotheses

#### Aims

To carry out a randomised controlled trial to evaluate a universal, school-based intervention to improve the mental health of secondary school students from low-income areas of Santiago. Specific aims are:

A) To quantify the effectiveness and cost-effectiveness of this intervention in reducing depressive symptoms among students.

B) To assess improvements in levels of functioning as secondary outcome measures and the role of mediating factors such as problem solving skills and dysfunctional negative thoughts.

C) To study factors influencing adherence and acceptability of this intervention.

#### Hypotheses

1) Students receiving the intervention will achieve lower scores (difference in mean of at least 0.4 standard deviations) in the depressive questionnaire in comparison to the control group 3 months after completing the course.

2) Symptomatic improvements achieved at 3 months will be maintained until the final assessment 12 months after completing the course.

3) The intervention will be more effective at 3 and 12 months follow up among students with higher depression scores at baseline.

4) Students receiving the intervention will show greater reductions in negative thoughts and improvements in problems solving skills than those in the control group.

### Design

This is a single-blind; cluster randomised controlled trial of a school-based, universal intervention to reduce depressive symptoms among junior secondary school students in Santiago, Chile. The cluster design was necessary to reduce contamination within schools.

#### Setting

Our sampling frame comprised all Municipal state-funded secondary mixed-sex schools in Santiago with more than one 1° Medio grade classes (n = 85). Overall there were approximately 349.588 students (age 14-18) receiving secondary education in Santiago in 2007, 79% of these in state funded schools. It was estimated that 87% of all youngsters aged 14-17 were attending secondary education in 2003 with low drop-out rates after the first year of schooling [41].

#### Population

All students attending 1° Medio grade (equivalent to 9 years of education) in the selected schools were eligible. We chose this class because it coincided with the beginning of the rise in the prevalence of depression; thus, it should reduce the potential 'floor-effect' and also be of interest to participants [24,25].

#### Inclusion and exclusion criteria

All students were invited to participate. Students in either trial arm with severe depressive episodes, according to BDI-II baseline assessment, but with no marked suicidal ideation were invited to attend but also encouraged to seek professional advice. Students with marked suicidal ideation at baseline in either group were referred for a clinical assessment in their primary care clinic. Students admitted to hospital for mental health reasons during the trial and those with serious alcohol/drug use were advised to continue with their prescribed treatment and in case of doubt referred for a clinical assessment. Most previous school studies show that less than 1% of students are excluded on the grounds mentioned above [21,24,26].

#### Recruitment/Allocation of schools

Randomisation took place once all schools were recruited and after the baseline in order to obtain balance with respect to size of schools, socio-economic deprivation within the area, and area of location of schools. This was achieved by calculating an imbalance statistic[42] for a large random sample of possible allocation sequences, then selection at random by an external statistician of one sequence from a subset with the most desirable balance properties. In those schools with more than 4 eligible classes only a maximum of 4 classes were randomly selected for the study.

#### Recruitment of students and consent process

Students and their parents were informed of the study. Parents were informed that the course was part of the school curriculum following approval by the school and educational authorities. Nonetheless parents were sent an assent form to provide an opportunity to request the withdrawal of their children from the study assessments. Children were asked to sign a written consent form. If a child expressed a desire not to participate in the course the school arranged alternative teaching activities for that pupil. Baseline information was collected from all students but those whose parents express their option for a withdrawal were not subsequently assessed.

#### Intervention

The intervention was based on a cognitive-behavioural therapy (CBT) model delivered to all students in the class during school hours. We developed the intervention after a period of 18 months of formative and pilot research. We included and adapted some ideas from several CBT depression prevention programmes for children within this age range; however, the intervention was developed specifically for this study.

The programme consisted of 11 weekly and 3 booster group sessions each lasting one hour. The number of sessions was comparable to most other similar studies with adolescents. In previous studies with other populations we have used even shorter group interventions with good results[37]. There was an introductory session, five sessions dealing with thought re-structuring, one session related to identifying emotions, three sessions of problem solving and one closing session with a revision of the learning and planning for the future.

Eight trios of trained research workers (psychologists, teachers, social workers, and others) delivered the intervention. These workers had a detailed manual specifying key learning points and objectives for each session and students received a similar but shortened workbook (available on request). Each session was delivered with the assisted of a power point presentation and a poster with key learning points was left in the classroom as well as a personal cards handed out with similar contents. Further material and examples were written in the students' workbooks. Methods included didactic sessions; small group and class interactive exercises. To ensure treatment integrity there was a detailed operational manual with checklists for therapist actions, training and supervision sessions, and a random sample of sessions were observed and evaluated by an independent rater. Two additional booster sessions were delivered at 2 and 7 months after the intervention was completed. All parents were offered one session of information on mental health but no information about specific youngsters was provided except when there was a marked clinical risk. Teachers were asked to assist with the discipline of the class in special circumstances. Previous studies suggested that the presence of teachers could inhibit students from sharing their experiences so we opted for allowing their presence only under special circumstances[24].

Facilitators received five days of training which covered the identification and management of mental health concerns, group management techniques as well as training to deliver the specific intervention. The intervention was fully manualised. During the course weekly supervision groups were provided for facilitators. Supervisors were experienced Senior Clinicians from the local team. They participated in the initial intervention training sessions so that they were familiar and knowledgeable about the intervention. One of the lead applicants offered support and advice to the group supervisors when needed.

#### Control

The control group received nothing other than the normal teaching activities and assessments. According to the school curriculum all classes receive one curricular hour weekly for counselling delivered by their head-teachers. We advised teachers to put more emphasis on emotional problems for 12 weeks giving more and better information, allowing students to exchange experiences and providing mutual support. If the active intervention proved to be more effective, we offered to implement the course in all control schools after completion of the trial.

#### Outcome measures

##### Primary

*Beck Depression Inventory II *(BDI-II)[43]as a continuous variable was defined as the primary outcome measure (baseline, 3 and 12 months). This is a brief and well-established depression questionnaire translated to different languages and used widely throughout the world. It has previously been used among adolescents in Chile[44] and in other Latin-American countries[45,46] showing good psychometric properties. It is self-completed which has the advantage of reducing potential observer bias since it is unlikely that observers will be completely blind to allocation. The BDI-II also provides a good measure of the cognitive changes expected to occur with the intervention.

##### Secondary

1) *Revised Child Anxiety and Depression Scale ***(**RCADS): This is an adaptation of the Spence Child Anxiety Scale (SCAS)[47] and intends to assess symptoms of DSM-defined anxiety disorders and major depression. The scale consists of 47 items that on the basis of exploratory factor analysis [48,49] are allocated to six subscales: social phobia (9 items), panic disorder (9 items), major depressive disorder (10 items), separation anxiety disorder (7 items), generalized anxiety disorder (6 items), and obsessive-compulsive disorder (6 items). Items have to be scored on a 4-point scale. RCADS subscale scores can be obtained by summing across relevant items. We excluded the depression and separation anxiety sub-scales because these were either covered by other scales or irrelevant to students of this age. 2) School records of academic performance: We will only use grades obtained through formal testing because these are standardised across schools.

##### Other assessments

1) *Measures of psychological functioning: Children's Automatic Thoughts Scale *(CATS)[50]. This self completed scale assesses a range of negative self statements in children and young people aged 7-16. For each item the child is asked to rate whether they have had a similar thought over the past week. Each item is rated as "not at all" (scores 0), "sometimes" (scores 1), "fairly often" (scores 2), "often" (scores 3) or "all the time" (scores 4). Confirmatory factor analysis identified 4 distinct but correlated factors relating to thoughts about physical threat, social threat, personal failure and hostility[51]. Internal consistency for the total score was high (Cronbach Alpha = 0.95) with acceptable test-retest reliability (0.79). The scale has been found to effectively discriminate between a community and clinical sample with the personal failure sub-scale being the strongest predictor of depressive symptoms [52]. The 10 item personal failure sub-scale will be used. 2) *The Short Form of the Social Problem-Solving Inventory Revised *(SPSI-R Short Form)[53] will be used to assess problem-solving dimensions. The SPSI-R Short Form is a 25-item self-report instrument that measures two adaptive problem-solving dimensions (positive problem orientation and rational problem solving) and three dysfunctional dimensions (negative problem orientation, impulsivity/carelessness style, and avoidance style). Each item is rated on a 5-point scale ranging from not at all true of me (0) to extremely true of me (4). Alpha coefficients for the subscales range from .72 to .85 [53]. Studies with the Spanish version of the SPSI-R Short Form confirmed its factor structure and obtained adequate alpha coefficients for the five subscales [54,55]. In the more recent study, alpha coefficients were .55, .73, .66, .70, and .69 for positive orientation, negative orientation, rational solving, impulsivity, and avoidance, respectively.

##### Further data collected included

1) Sex and date of birth, 2) family and socio-economic circumstances extracted from school records, 3) Previous personal history of mental problems, 4) use of drugs and/or alcohol, 5) contacts/consultations with health or psychological care professionals, 6) Self-harm questionnaire, and 7) Class climate observation scale

#### Methods for data collection

Independent researchers blind to allocation undertook the 3 month follow-up and are currently undertaking the 12 month assessment. Researchers are constantly rotated among schools. All assessors received a full day of training to ensure data collection was fully standardised. It was virtually impossible to blind assessors completely in a study of this kind and individual assessments are unfeasible so we relied largely on self-reports complemented with teachers' brief reports. Assessors were asked to guess the group to which they belong to check the efficacy of allocation concealment.

#### Process evaluation

Number of sessions attended, professional referrals to outside agencies and other treatment received were recorded. Activities undertaken in the control group were carefully recorded. These data will be used to generate unit costs for the economic analysis. We did not anticipate any possible adverse outcome directly related to the intervention but we kept a record throughout the study of any suicidal attempts or cases which required psychiatric consultations. Whenever researchers doing the fieldwork received any such information this was entered in a central log diary. We also asked the school to inform us of any such events. If there were any potentially adverse events reported the Management Committee assessed the situation to determine if there was any connection with the study.

A semi-structured assessment was undertaken at the end of the programme to assess participant's perception of: (a) the intervention, usefulness, satisfaction, what they had learned and evidence of on-going skill usage. Analysis of this data has already been undertaken but remains unpublished. At the end of the programme there was also a focus group with a sample of facilitators randomly selected to assess their views of the programme. Interviews were tape recorded and covered a range of factors including participant engagement, school/class teacher support, leader confidence and effectiveness in delivering the programme and perception of participant usefulness. A brief survey of all facilitators was also conducted.

#### Sample size

Studies of similar school-based interventions report effect sizes of at least 0.4 standard deviations (SDs) using brief symptom questionnaires as proposed here. This is a conservative estimate based on studies comparing interventions against placebo-controlled groups with several non-specific therapeutic components. The potential benefits for society of a shift in the mean of this magnitude are substantial: a reduction in the prevalence of diagnosed common emotional disorders from 10% to 4.7% and important benefits for a large proportion of students with milder symptoms[56]. We calculated an intra-cluster correlation coefficient (ICC) of 0.041 (95% CI 0.036-0.046) for 'negative emotionality' using a dataset comprising 58,000 students in 1396 secondary schools in Chile. To detect a difference of 0.4 SDs with 90% power and two-sided 1% alpha would require a total of N = 376 for analysis. However this number invited must be inflated to allow for clustering, non-consent and loss to follow up. There were 85 state-funded mixed-sex schools in the greater Santiago area with >1 class per year group. We randomly selected four classes for study in those schools with more than four per year group. Therefore schools participating in the study had 2, 3 or 4 classes in the trial, yielding a mean year group size of 125 (SD 40), and a mean cluster size for analysis of 80 (SD 26) assuming 80% consent and retention rates. The sample was stratified according to number of classes in each school (2/3 vs 4 classes) and socio-economic status of the area where the school was located (tertiles). Schools were proportionately randomly selected to ensure all strata were represented. Using Eldridge et al's[57] formula for inflation of sample size in cluster randomised trials with unequal cluster sizes*, we calcualted needed to invite 2634 students from 20.3 schools in order to maintain 90% power for the primary analysis. We therefore aimed to recruit and randomise 22 schools. A list of 22 schools representing all strata was chosen. When a school from this list refused to take part, we selected the first available school within the appropriate stratum within our Reserve List.

#### Data analysis

Analysis is due to start shortly and presentation of data will be in accordance with CONSORT guidelines, with the primary comparative analysis being conducted on an intention-to-treat basis and due emphasis placed on confidence intervals for the between-arm comparisons. Descriptive school-level and student-level data will be used to ascertain any marked imbalance between the arms at baseline. The primary analysis will employ multivariable regression to investigate differences in mean symptom scores (primary outcome measure) between groups at 3 months follow up, adjusting for stratification variables and baseline outcome variable scores, and taking account of the hierarchical nature of the data. Sensitivity analysis making different assumptions will be conducted to investigate the potential effects of missing data. Secondary analyses will include: 1) repeating the primary analysis adjusting also for any variables exhibiting marked imbalance at baseline to examine whether this influences the findings; 2) comparison of changes in primary outcome measure between groups at 12 months (persistence of improvement); 3) similar analyses for secondary outcomes (where p-values will be adjusted for multiple testing); 4) investigation of process measures such as the number of sessions attended. Finally appropriate interaction terms will be entered into the primary regression analyses in order to conduct pre-specified subgroup analyses according to symptom severity and the impact of any additional treatment received. Since the trial is powered to detect overall differences between the groups rather than interactions of this kind, the results of these essentially exploratory analyses will be presented using confidence intervals and p-values, and interpreted with due caution. Qualitative data will be analysed thematically using the latest version of NUD*IST a software for analysing text-based data. Tapes will be transcribed and codebooks will be generated.

The economic analysis will take a societal perspective, with direct and indirect costs, computed at completion of the course, 3 and 12 months later. Direct costs in the intervention group will include fixed and recurrent costs. Fixed costs will be allocated across all randomised participants but variable costs will depend on individual use of resources. These costs will be related to changes in the primary outcome. Cost-benefit modelling will be used in the analysis. Confidence intervals and acceptability curves for cost-effectiveness ratios will be derived (using bootstrapping techniques) in order to show the probability of any cost-effective advantages for the component interventions at a range of 'willingness to pay' threshold levels.

#### Research Governance and Ethics

*Trial Management*: The study complied and was conducted in accordance with local Research Governance requirements. There were three main committees: 1) Trial Steering Committee (TSC): This committee met once every two years. Its role was to monitor and supervise the progress of the trial towards achieving its goals; to advise the investigators in general scientific and management issues; and to ensure that there were no major deviations from the study protocol. The Lead applicant informed the Chair of the TSC who was allowed to call additional teleconference meetings when there were matters arising from the conduct or management of the trial that required their advice. 2) Trial Management Group: A separate Trial Management Group was established to oversee the operational running and progress of the project. This met monthly and included the senior researchers and other co-applicants as appropriate. There was a monthly teleconferencing with the main applicant. 3) Finally, there was an independent Data Monitoring and Ethics Committee. This monitored data and advised the TSC on whether there were any ethical or safety reasons why the trial should not continue.

##### Ethics

Full ethical approval was obtained from the local Committee (Hospital Clinico Universidad de Chile). Evaluation of opt-in and opt-out recruitment strategies suggested that opt in strategies resulted in lower recruitment rates and healthier participants. Some authors have suggested that opt-out approaches should be the default recruitment strategy for interventions that pose a low risk to participants. The participants in this study were not referred patients, the intervention was low risk, and as such we used an opt-out approach. At the start of the project a letter was sent to the carers of all eligible young people informing them about the study. The letter therefore informed carers that they could opt out of the assessments if they did not wish their child to complete the questionnaires. In addition, written child consent was obtained before completing the questionnaires i.e. dual carer/child consent/assent was required.

## List of abbreviations used

YPSA: Yo Pienso, Siento, Actuo; CBT: Cognitive-Behavioural Therapy; IPT: Interpersonal Psychotherapy; SD: standard deviation; BDI-II: Beck Depression Inventory II; RCADS: Revised Child Anxiety and Depression Scale; SCAS: Spence Child Anxiety Scale; DSM: Diagnostic and Statistical Manual; CATS: Children's Automatic Thoughts Scale; SPSI-R: Short Form of the Social Problem-Solving Inventory Revised; ICC: intra-cluster correlation coefficient; TSC: Trial Steering Committee

## Competing interests

The authors declare that they have no competing interests.

## Authors' contributions

Ricardo Araya, Alan Montgomery, S Noble, P Stallard, D Gunnell, G Rojas and R Fritsch conceived the study and led the bid to secure funding for this work. All of them contributed to the development of the protocol and were involved in managing and advising on the project. Vania Martinez, Sergio Barroilhet, Paul Vohringer, and Jorge Gaete contributed to the development of the protocol and the drafting of this paper. All authors read and approved the final manuscript.
